# Cognitive Processes during Recovery: Moving toward Personalized Spine Surgery Outcomes

**DOI:** 10.3390/jpm12101545

**Published:** 2022-09-20

**Authors:** Carolyn E. Schwartz, Bruce D. Rapkin, Katrina Borowiec, Joel A. Finkelstein

**Affiliations:** 1 DeltaQuest Foundation, Inc., Concord, MA 02111, USA; 2 Departments of Medicine and Orthopaedic Surgery, Tufts University School of Medicine, Boston, MA 02111, USA; 3 Department of Epidemiology & Population Health, Albert Einstein College of Medicine, Bronx, NY 10461, USA; 4 Department of Measurement, Evaluation, Statistics & Assessment, Boston College Lynch School of Education and Human Development, Chestnut Hill, MA 02467, USA; 5 Department of Surgery, University of Toronto, Toronto, ON M4N 3M5, Canada; 6 Division of Orthopedic Surgery, Sunnybrook Health Sciences Centre, Toronto, ON M4N 3M5, Canada; 7 Division of Spine Surgery, Sunnybrook Health Sciences Centre, Toronto, ON M4N 3M5, Canada

**Keywords:** spine surgery, quality of life, disability, Oswestry Disability Index, mental health functioning, cognitive appraisal

## Abstract

This paper focuses on a novel application of personalized medicine: the ways one thinks about health (i.e., appraisal processes) as relevant predictors of spine-surgery response. This prospective longitudinal cohort study (n = 235) investigated how appraisal processes relate to outcomes of spinal decompression and/or fusion surgery, from pre-surgery through one-year post-surgery. Patient-reported outcomes assessed spine-specific disability (Oswestry Disability Index (ODI)), mental health functioning (Rand-36 Mental Component Score (MCS)), and cognitive appraisal processes (how people recall past experiences and to whom they compare themselves). Analysis of Variance examined the appraisal-outcomes association in separate models at pre-surgery, 3 months, and 12 months. We found that appraisal processes explained less variance at pre-surgery than later and were differentially relevant to health outcomes at different times in the spine-surgery recovery trajectory. For the ODI, recall of the seriousness of their condition was most prominent early in recovery, and comparing themselves to positive standards was most prominent later. For the MCS, not focusing on the negative aspects of their condition and/or on how others see them was associated with steady improvement and higher scores at 12 months. Appraisal processes are relevant to both spine-specific disability and mental-health functioning. Such processes are modifiable objects of attention for personalizing spine-surgery outcomes.

## 1. Introduction

Personalized medicine is a medical model that separates people into different groups on the basis of characteristics deemed relevant to their predicted response or risk of disease [1]. It often focuses on “hard” parameters such as genetic or epigenomic makeup, other biomarker information, and clinical information [2,3]. Considering “softer” parameters is warranted, nonetheless [4,5,6]. Research over the past two decades has documented that cognitive appraisal processes—the ways one thinks about health—explain substantial variance in health, group differences in health outcomes, and adaptation to changing health [7,8,9,10,11,12].

In the context of spine-surgery outcomes, the importance of cognitive appraisal processes is increasingly documented. It is known, for example, that patient expectations can affect the perceived outcome to treatment [13], and that patients’ interpretation of their symptoms and their trajectory will impact their pain and their response to treatment [14]. Directly addressing pain sufferers’ cognitive processes, beliefs, and expectations has been posited to increase our understanding of outcome disparities between patients with the same diagnosis and treatment [14].

In two recent analyses of factors that help predict postoperative outcomes following orthopaedic surgery, patients’ cognitive appraisal processes were among the few retained variables predicting functional outcomes [15,16]. Indeed, patient’s use of specific cognitive processes pre-surgery explained 31–40% of the variance in reported pain and functioning post-surgery [15,16]. Appraisal processes in quality-of-life (QOL) outcomes assessment provide insights into broader cognitive, social, and affective processing of health [17]. How people appraise QOL relates to how people self-monitor their health status; their ability to self-regulate health behavior; and their sense of control over their health outcomes.

Appraisal processes differ both across individuals and within individuals at different points in time and with respect to specific contexts [18,19]. Changes in appraisal processes over time can lead to response-shift effects [17,20], if these changes explain variance in the discrepancy between expected and observed QOL [17,20]. Appraisal measures are idiometric, in that they assess thought processes that are contingent on circumstances, and thus do not reduce to simple scale scores that are consistent across samples [21]. Consequently, one must examine appraisal processes individually (i.e., as separate items) [21]. As a main effect, appraisal can highlight underlying differences in how people think about QOL that impact or obfuscate score differences between groups [22]. As a time-varying effect, appraisal changes over time may reflect adaptation to changing health [20]. Appraisal assessment can help to portray individual differences in terms that depict how QOL concerns and priorities influence their evaluation of physical and mental health [22].

Cognitive appraisal processes thus would have clear relevance and implications to personalized medicine. An individual’s focus on specific cognitive appraisal processes might be associated with an increased risk of worse outcome or recovery trajectory, and other specific processes might be associated with greater therapeutic benefit and faster recovery. Clinicians could utilize information about cognitive appraisal processes associated with worse or better outcomes by encouraging patients prior to surgery or at relevant timepoints after surgery to focus on more adaptive appraisals to facilitate unhindered recovery. Identifying the appraisal processes that play important roles in outcomes at various time points following surgery would be an important foundation for such a personalized medicine approach.

The present study aimed to investigate appraisal processes explained variance in spine-surgery outcomes over the course of the recovery trajectory, from pre-surgery through one-year post-surgery. It examined two types of patient-reported outcomes (PRO): one that showed clear responsiveness to spine surgery (i.e., clear improvement on the PRO over time after surgery), and one that showed minimal responsiveness (i.e., PRO remained relatively stable over time after surgery). We hypothesized that appraisal processes would help to explain differences over time in the responsive outcome, and that underlying differences in appraisal processes used would clarify lack of change on the non-responsive outcome. We focused on two types of appraisal processes: how people recall past experiences (sampling of experience) and to whom people compare themselves (standards of comparison).

## 2. Materials and Methods

### 2.1. Sample and Design

This prospective longitudinal cohort study included adults who were recruited from an active spine surgery practice from a Canadian academic teaching hospital from December 2008 through September 2021. Eligibility criteria included being over the age of 18 and having undergone elective spinal decompression and/or fusion surgery. Exclusionary criteria entailed having had prior lumbar surgery at the same level, or being unable to understand and complete the English survey-related documents. Diagnoses were disc herniation, radiculopathy/sciatica, spinal stenosis with neurogenci claudication, and degenerative spondylolisthesis. The similarity in these pathologies is the leg-dominant pain. The decision as to whether to do decompression and fusion or just decompression alone followed evidence-based guidelines [23,24]. All patients provided written informed consent prior to completing any questionnaires. Data were collected online or by mail at pre-surgery and at approximately 3 months, and 12 months post-surgery using a secure, Health Information Portability and Accountability Act (HIPAA)-compliant interface [25]. The study was reviewed and approved by the Sunnybrook Health Centre Research Ethics Board.

### 2.2. Measures

Spine-specific disability was measured using the 10-item Oswestry Disability Index (ODI) [26]. This ten-item measure is the most commonly used tool in both operative and non-operative spine patient cohorts. The ODI assesses the level of pain and interference with physical activities, sleeping, personal care, social life, sex life, and travelling. Each item is scored from 0 to 5 (0 severe disability to 5 which is little disability). The ODI yields a spine-specific disability (scale) score between 0 and 100. For the present work, we recoded the ODI so that higher scores reflect lower pain-related interference with ADLs to reduce confusion by being consistent with the other measure used in the present analysis.

The Mental Component Score (MCS) of the Medical Outcomes Study Short-Form (Rand-36) [27,28,29] assessed mental-health functioning. This score is created by summing the eight standardized domain scores weighted by factor score coefficients that lend the most weight to the mental health, role emotional, social functioning, and vitality domains [30,31]. The scores are then transformed to a norm-based T-score, with a mean of 50 and standard deviation of 10 [31]. The population norm is a score of 50 [30].

Cognitive appraisal processes have been well studied in a diverse group of medical illnesses and the Quality of Life Appraisal Profilev2 Short-Form (QOLAPv2-SF) [32] has been validated for this purpose. The items contained in the QOLAPv2-SF were derived from a series of studies in medically ill patient groups, studying the ways people think about QOL starting with open-text qualitative data to closed-ended quantitative data with data from over 6400 patients [21,32]. Items utilized a 5-point rating scale (Never, Rarely, Sometimes, Often, Always), with higher values assigned to more endorsement. QOLAP_v2_-SF contains four domains, two of which were the focus of the present work. The 14 *Sampling-of-Experience* items query how people recall or remember past experiences when responding to QOL measures. The 8 *Standards-of-Comparison* items query to whom or what the individual compares themself to when thinking about QOL. (As the QOLAP is a copyrighted measure, its items cannot be published in the present work’s tables. Interested readers are invited to contact the first author for more information about and access to the measure).

To describe the sample, demographic characteristics were collected, including age, gender, smoking status, and education. Clinical data included diagnosis, primary procedure, pain medicine frequency, and comorbidities, the latter of which was assessed using the Self-Administered Comorbidity Questionnaire [33].

### 2.3. Statistical Analysis

Descriptive statistics were used to summarize the study sample. Analysis of Variance (ANOVA) models examined the association between QOLAP_v2_-SF items (independent variables) and ODI or MCS (dependent variables in separate models). We tested each time window specified in the study protocol separately: pre-surgery, 3 months, and 12 months. Appraisal items were also treated as categorical variables. This treatment enables detection of non-linear relationships and does not presume equal intervals across response options on the appraisal items. We collapsed the bottom and top two response options (i.e., never and rarely were combined; often and all of the time were combined; sometimes remained its own category). Pearson correlations investigated the association between ODI and MCS at baseline, 3 months, and 12 months post-surgery. To guide interpretation of patterns in explained variance over study time windows, Cohen’s published cut-offs for explained variance (eta^2^) were used [34]. Software data were analyzed using IBM SPSS version 26 [35].

## 3. Results

### 3.1. Sample

The study sample included 235 people who underwent spine surgery. Most (65%) patients received a laminectomy/discectomy; 11% received instrumentation/fusion; and 20% instrumentation/fusion and laminectomy/discectomy (Table 1). Table 1 provides descriptive statistics on the sample.

### 3.2. Change in Outcomes over Time

**Figure 1:**
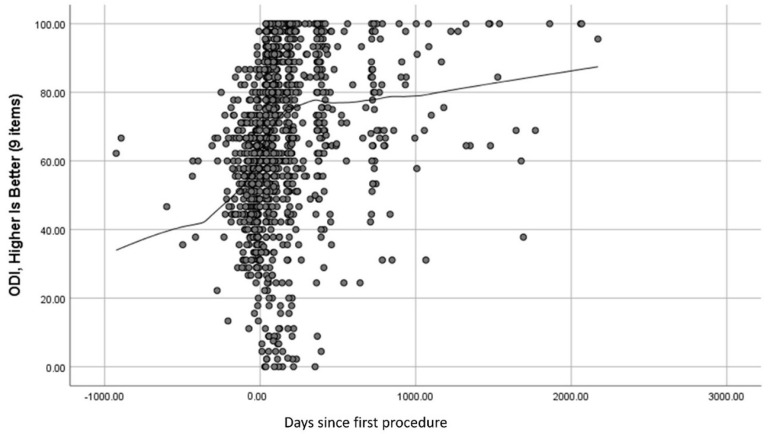
Scatter Plot of ODI Scores Over Time.

**Figure 2:**
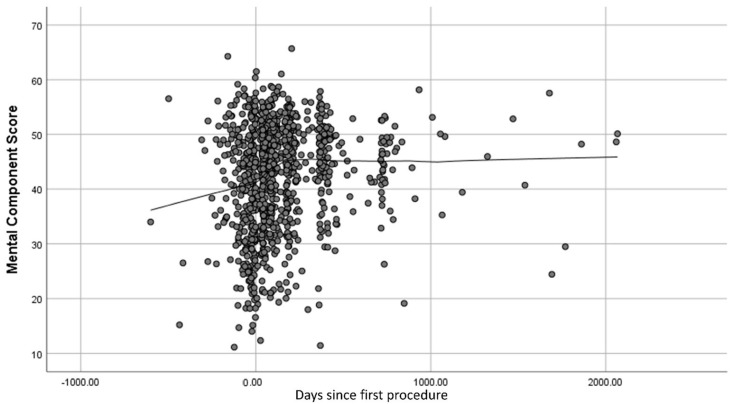
Scatter Plot of MCS Scores Over Time.

Figure 1 and Figure 2 show scatter plots of ODI and MCS scores over time. Locally Weighted Scatterplot Smoothing (lowess) lines show the trends over time on these two outcome variables. The ODI appears throughout follow-up to be responsive to spine surgery (i.e., scores reflect improvement post-surgery), while the MCS was responsive in the first 12 months post-surgery but plateaued after that. Additionally, the sample was lower than the population norm on this mental-health functioning score throughout follow-up, which is about 52 in a similar age cohort [30].

### 3.3. Association of Appraisal with ODI

Table 2 provides the eta^2^ estimates for ODI predicted by QOLAP_v2_-SF items at each time window. Conditional formatting shows the effect size (ES) magnitude using Cohen’s criteria as cut-offs [34]. Sampling of Experience items explained slightly higher amounts of variance at 3 months post-surgery compared to pre- or 12 months post-surgery (average eta^2^ = 0.02, 0.04, and 0.03, respectively), with several items within this domain explaining medium ES associations (Table 2). In contrast, Standards of Comparison items explained more variance at 12 months post-surgery (average eta^2^ = 0.03, 0.01, and 0.05, respectively), with several items within this domain explaining medium ES associations (Table 2).

There were three general patterns of association between appraisal and spine-specific disability over time (Table 2). First, appraisal processes explain less variance (i.e., are less associated with spine-specific disability) at pre-surgery than at subsequent time points. Second, some items explained more variance *early in the recovery trajectory* (i.e., at 3 months, e.g., balancing the positives and the negatives, focusing on their spinal condition, trying to communicate the seriousness of their condition). All of these were Sampling of Experience items. Third, other appraisal items became more important (i.e., explained more variance) *later in the recovery trajectory* (e.g., comparing themselves to others with a spinal condition, without any health limitations, their perfect-health ideal, the way others see them). All of these were Standards of Comparison items.

**Figure 3:**
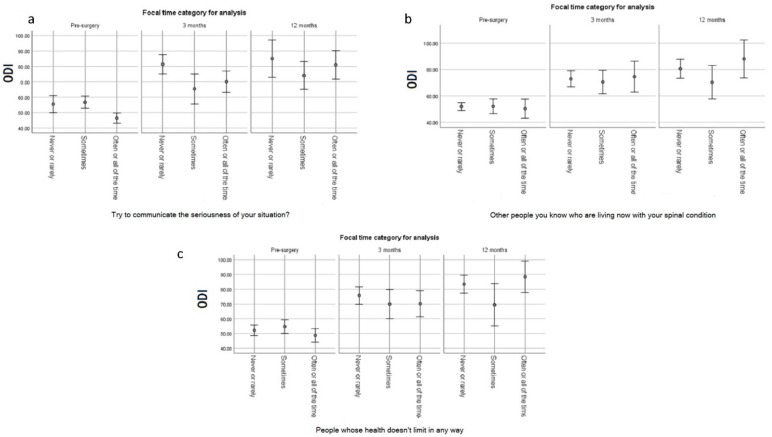
Relationship of Appraisal with ODI Over Time (**a**–**c**).

Figure 3a–c illustrate changes over time in the relationships between selected appraisal processes and the ODI. For the Sampling of Experience item that explained the most variance early in the trajectory, the plot illustrates that those who never or rarely sampled experiences to emphasize the seriousness of their condition improved quickly (by 3 months post-surgery) and maintained this improvement at 12 months (Figure 3a). They also achieved a higher ODI than the others. In contrast, those who often or all the time emphasize the seriousness of their condition started out worse than the others and had a slower and more gradual improvement trajectory (Figure 3a).

For the two Standards of Comparison items shown in Figure 3b,c, the association with ODI was negligible or small early in the recovery, but substantial at 12 months. In both cases, people who sometimes compared themselves to others with a spinal condition or to others with no health limitations reported the lowest ODI at 12 months compared to the others. In contrast, those who often or always used these two standards of comparison had a steady improvement trajectory and achieved a higher ODI than the others. Those who never or rarely used such standards of comparison ended up with ODI scores lower than the often/always group but higher than the sometimes group.

### 3.4. Association of Appraisal with MCS Mental-Health Functioning

**Figure 4:**
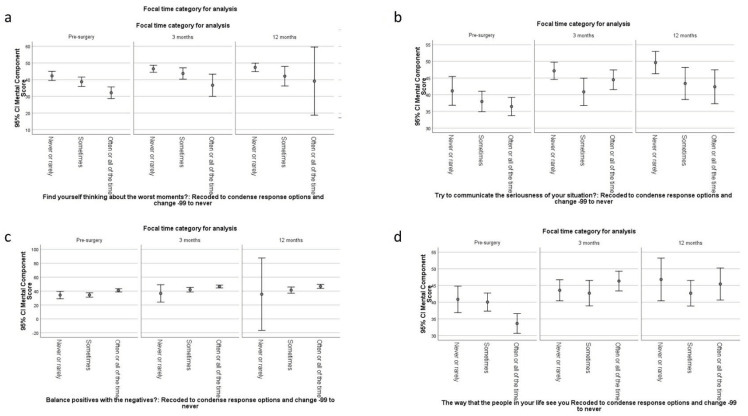
Relationship of Appraisal with MCS Over Time (**a**–**d**).

There was a general pattern of association between appraisal and mental-health functioning over time: the two constructs became more strongly associated with time. Whereas about half of the appraisal processes explained small ES variance with MCS pre-surgery, these associations often grew to medium ES at 3- and 12 months post-surgery (Table 3). Further, this pattern was particularly notable for Sampling-of-Experience appraisal processes related to focusing on the negative aspects of their condition. For example, those who never or rarely focused on the worst moments started out and ended up with higher MCS scores than the others and showed a steady upward trajectory (Figure 4a). This was also the case for those who never or rarely focused on communicating the seriousness of their situation (Figure 4b). Focusing on balancing the positives and the negatives maintained a steady medium ES throughout follow-up (Table 3), with those who often or always endorsed this approach scoring slightly higher than the others (Figure 4c).

Standards-of-comparison appraisals explained increasing variance in mental-health functioning with time (average eta^2^ = 0.03, 0.04, and 0.05, for pre-, 3 and 12 months post-surgery, respectively (Table 3). At 12 months follow-up, comparing oneself to others with a spinal condition, those without health limitations, and the kind of life one is really working for all explained medium ES variance in MCS (Table 3). In contrast, comparing themselves to how people in their life see them was more important for mental health at pre-surgery and its importance declined over time. In general, those who never or rarely compared themselves to how others saw them reported better mental health at all time points but less change over time (Figure 4d). In contrast, those who often or always compared themselves to how others saw them improved quickly (by 3 months post-surgery) and maintained this improvement at 12 months (Figure 4d).

Pearson correlations between the ODI and MCS scores revealed a negligible association at baseline, small ES correlations at 3 months post-surgery, and medium ES correlations at 12 months post-surgery (r = 0.09, 0.21, and 0.39, respectively; data not shown). Thus, when spine-specific disability was at its worst (i.e., prior to surgery), there was no association with mental-health functioning. As it improved, it was increasingly associated with better mental health.

## 4. Discussion

The present study revealed that appraisal processes are relevant to health outcomes after spine surgery, with different processes coming into play at different points in the recovery trajectory. Cognitive appraisal is an independent variable or a patient-specific characteristic that will have impact on outcomes after spine surgery. It is thus a relevant consideration for personalized medicine.

As hypothesized, the two outcomes that were the focus of the present work because they demonstrated variability in the responsiveness to spine surgery, also differed in the magnitude of the role played by appraisal. With regard to responsiveness, the ODI showed great improvement over the first 12 months after surgery and continued an upward trend thereafter. In contrast, the MCS showed notable but lesser improvement than the ODI over the first 12 months after surgery and then plateaued. These findings were similarly seen in a total hip arthroplasty population between a disease-specific outcome measure (the Hip Disability and Osteoarthritis Outcome Score [36]) and the MCS [37]. Of note, the relationship between these two outcomes varied over follow-up: they were uncorrelated at baseline and became more strongly correlated over time. Slower ODI improvement may be a drag on mental-health functioning. Additionally, the impact of poor pre-morbid (pre-surgery) mental health on ODI may be overpowered at baseline by pain, but re-asserts itself as ODI improves. Both processes may be happening. These patterns may relate to our findings that recall (Sampling of Experience) mattered earlier, while standards (Standards of Comparison) mattered more later. Nonetheless, the average MCS score remained well below the population norm for the sample’s age group.

Spine-specific disability and mental-health functioning also differed in the magnitude of the role played by appraisal processes: more variance in MCS was explained by appraisal on average than for the ODI. These findings suggest that appraisal processes may play a role in helping individuals maintain their mental-health functioning whereas such processes play less of a role for spine-specific disability. Conversely, appraisal may also relate to what is attenuating improvement in mental-health functioning. In other words, there may be ways of thinking about QOL that enable better mood and well-being. Future research might investigate who are those people who did not get better despite their pain getting better.

As described above, although patients’ mental health improved, it never achieved what would be expected for their cohort. It would be worthwhile to examine system, clinical, and person factors that might relate to higher-than-norm versus lower-than-norm mental-health levels. For example, does delay between diagnosis and surgical intervention predict mental-health functioning (system factor)? Are some conservative approaches to pain management during the period of waiting for surgery associated with better mental health compared to other conservative approaches (clinical factor)? Do people who continue to engage in exercise report better mental health despite similar levels of spine-specific disability (person factor)? A comprehensive examination of such diverse factors might lead to a better understanding of how to improve this important facet of QOL from pre-surgery through long-term follow-up.

## 5. Clinical Implications

A growing body of evidence supports the clinical, economic, and psychology benefit of preoperative education focused on improving patient knowledge, their feeling prepared, reducing negative thinking, and increasing levels of physical activity after spine surgery [38]. The clinical implications of this work can be applied to already established perioperative pathways. Enhanced Recovery After Surgery (ERAS) is a multimodal approach that is increasingly used in the care of surgical patients [39]. ERAS pathways for spine surgery reduce lengths of stay, accelerate return of function, minimize postoperative pain, and lower costs [40], but fail to address many of the social and psychological aspects that are important to patients undergoing surgery. A preoperative physiotherapy intervention was reported to help patients change specific negative cognitive factors, such as fear of movement and pain catastrophizing, and to increase their functional self-efficacy [41]. This literature thus suggests that cognitive processes are modifiable and worthy objects of intervention.

Preoperative identification of at-risk patients would be the first step in addressing this. The Sampling-of-Experience and Standards-of-Comparison sections of the QOLAP_v2_-SF could be used as a screening tool. For example, if someone is overly focused on the seriousness of their situation, it may mean that additional problems are present (i.e., things really are more serious) or that an individual’s expectations are off (e.g., they expect a recovery that is faster than realistic). Directly addressing the individual’s context and their expectations could be done with a “prehabilitation” type approach [42,43,44]. As part of this prehabilitation, clinicians from a range of disciplines (e.g., physicians, nurses, physical and occupational therapists, social workers, and counselors) could work together to prepare patients for surgery.

## 6. Limitations

The present work has the advantage of a longitudinal design with data collected at clinically relevant milestones with regard to spine-surgery recovery. Its limitations must, however, be acknowledged. First, the QOLAP_v2_-SF domains were analyzed at the item level rather using data-reduction techniques. This is consistent with standards for analysis of idiometric measures [21]. This led to a number of statistical comparisons which could have inflated the Type I error rate. Second, treating appraisal responses as categorical rather than continuous also increased the size of the eta^2^. We mitigated both of these concerns by focusing on interpreting mid-to-large ESs, rather than small ESs or statistical significance. Third, it is possible that participant attrition biased the study findings. In other words, people who dropped out of the data set might have been those doing worst. We do not believe this is the case, based on other work by our group on the same data set: we found that an analysis of attrition did not support this hypothesis [45]. Fourth, age may influence results. Future research might consider differences in appraisal patterns by age in a larger sample that adequately represents different developmental stages of adulthood. Finally, the sample was predominantly White and educated, thus limiting our study’s generalizability to other race/ethnicity groups and people with less education and other resources [46]. Future work is needed to replicate the analyses in a larger, more diverse sample.

## 7. Conclusions

In summary, cognitive appraisal processes are relevant to personalized medicine. More appraisal processes explained substantial variance in mental-health functioning than in spine-specific disability. Appraisals focused on difficult life challenges were associated with worse outcomes overall, and focusing on comparing themselves to how others see them was associated with worse mental health functioning prior to surgery but associated with a faster recovery. The clinical implications of this work may involve practical support for life challenges, and emotional support to reframe dependency during recovery so that it is experienced as less worrisome. Clinicians might explicitly discuss with patients the importance of how they think about health during the first year after spine surgery, and the importance of considering contextual demands in coping with the long-term recovery trajectory after spine surgery.

## Figures and Tables

**Table 1:** Descriptive Statistics of Study Sample † (N = 235).

Variable	Mean	Standard Deviation (SD)
**Age**	59.5	16.0
Range	18–88	
Comorbidities *, out of 12 presented	0.8	1.2
Range	0–7	
**Follow-up Time in Days**		
At 3 months	88.6	8.3
Range	75–105	
At 12 months	369.3	5.8
Range	360–380	
**Gender**		
Male	120	51%
Female	114	49%
Prefer not to answer	1	0%
Diagnoses *		
*Disc Herniation*		
Yes	75	32%
No	127	54%
Missing	33	14%
*Radiculopathy/sciatica*		
Yes	28	12%
No	174	74%
Missing	33	14%
*Spinal stenosis with neurogenic claudication*		
Yes	129	55%
No	73	31%
Missing	33	14%
*Spondylolisthesis (Lytic)*		
Yes	9	4%
No	193	82%
Missing	33	14%
*Spondylolisthesis (degenerative)*		
Yes	42	18%
No	160	68%
Missing	33	14%
**Primary Procedure**		
Lami/disc	152	65%
Instr/fusion along	27	11%
Instr/fusion w lami disc	46	20%
Missing	10	4%
**Pain Medicine Frequency**		
Not at all	27	11%
Once a week	41	17%
Once every couple days	15	6%
Once or twice a day	55	23%
3 or more times a day	63	27%
Missing	34	14%
Specific Comorbidities * (back pain excluded)		
Anemia	3	1%
Cancer	7	3%
Depression	21	9%
Diabetes	18	8%
Heart disease	14	6%
High blood pressure	52	22%
Kidney disease	5	2%
Liver disease	2	1%
Lung disease	5	2%
Osteoarthritis, degenerative arthritis	32	14%
Ulcer or stomach disease	4	2%
**Smoking Status**		
Never smoked/used tobacco	108	46%
Used to smoke/use tobacco	85	36%
Currently smoke/use tobacco	22	9%
Missing	20	9%
**Level of Education**		
Less than high school	16	7%
Graduated from high school or GED	29	12%
Some college or technical school	31	13%
Completed technical school (college)	7	3%
Graduated from college	53	23%
Postgraduate school or degree	45	19%
Missing	54	23%

† Data reflect baseline values for all variables except follow-up time in days. * For these topics, a non-response was counted as the absence of the event in question (no disease, no use of the medication, etc.).

**Table 2:** ODI: Eta^2^ in One-Way ANOVA.

	Time Window		
QOLAP_v2_-SF Item	Pre-Surgery	3 Mos. Post Surgery	12 Mos. Post Surgery
**Sampling of Experience Items**			
Worst moments	0.06	0.00	0.05
Emphasize positive	0.01	0.01	0.04
Recent few weeks	0.00	0.07	0.02
Relevant past 3 mo.	0.01	0.02	0.04
Balance positive/negative	0.01	0.06	0.03
Recent flare-ups	0.01	0.05	0.01
Future	0.00	0.03	0.02
Focus on spinal condition	0.01	0.08	0.06
Relationships	0.03	0.02	0.03
Doctor told	0.03	0.02	0.01
Only for survey	0.02	0.00	0.01
First reaction	0.01	0.06	0.00
Not complain	0.02	0.01	0.00
Seriousness	0.10	0.08	0.04
**Standards of Comparison Items**			
Others with spinal condition	0.00	0.00	0.06
Healthy others	0.02	0.02	0.13
Doctor said	0.02	0.03	0.03
Perfect health	0.04	0.00	0.08
Life working for	0.06	0.02	0.00
Way others see you	0.02	0.00	0.08
People your age	0.02	0.01	0.00
Time before health condition	0.02	0.03	0.02
*Sampling of Experience Average*	0.02	0.04	0.03
*Standards of Comparison Average*	0.03	0.01	0.05

Conditional formatting reflects effect-size magnitude per Cohen. Light blue shading reflects small ES, and light green reflects medium ES.

**Table 3:** MCS: Eta^2^ in One-Way ANOVA.

	Time Window		
QOLAP_v2_-SF Item	Pre-Surgery	3 Mos. Post Surgery	12 Mos. Post Surgery
**Sampling of Experience Items**			
Worst moments	0.14	0.14	0.11
Emphasize positive	0.05	0.10	0.07
Recent few weeks	0.02	0.04	0.09
Relevant past 3 mo.	0.02	0.00	0.07
Balance positive/negative	0.10	0.12	0.12
Recent flare-ups	0.05	0.05	0.06
Future	0.02	0.01	0.10
Focus on spinal condition	0.00	0.08	0.04
Relationships	0.02	0.04	0.05
Doctor told	0.01	0.01	0.01
Only for survey	0.02	0.03	0.02
First reaction	0.01	0.03	0.01
Not complain	0.03	0.07	0.04
Seriousness	0.03	0.07	0.12
**Standards of Comparison Items**			
Others with spinal condition	0.04	0.03	0.07
Healthy others	0.03	0.00	0.08
Doctor said	0.01	0.01	0.01
Perfect health	0.03	0.05	0.04
Life working for	0.01	0.00	0.08
Way others see you	0.09	0.03	0.03
People your age	0.03	0.07	0.02
Time before health condition	0.02	0.10	0.02
*Sampling of Experience Average*	0.04	0.06	0.07
*Standards of Comparison Average*	0.03	0.04	0.05

Conditional formatting reflects effect-size magnitude per Cohen. Light blue shading reflects small ES, light green reflects medium ES, and dark green reflects large ES.

## Data Availability

The study data are confidential and thus not able to be shared.

## References

[1] Charlton V. (2015). Stratified, personalised or P4 medicine: A new direction for placing the patient at the centre of healthcare and health education. Forum Academy of Medical Sciences.

[2] Cirillo D., Valencia A. (2019). Big data analytics for personalized medicine. Curr. Opin. Biotechnol..

[3] Goetz L.H., Schork N.J. (2018). Personalized medicine: Motivation, challenges, and progress. Fertil. Steril..

[4] Lorenzo-Luaces L., Peipert A., Romero R.D.J., Rutter L.A., Rodriguez-Quintana N. (2020). Personalized Medicine and Cognitive Behavioral Therapies for Depression: Small Effects, Big Problems, and Bigger Data. Int. J. Cogn. Ther..

[5] Barrecheguren M., Bourbeau J. (2018). Self-management strategies in chronic obstructive pulmonary disease: A first step toward personalized medicine. Curr. Opin. Pulm. Med..

[6] Hollister B., Bonham V.L. (2018). Should electronic health record-derived social and behavioral data be used in precision medicine research?. AMA J. Ethics.

[7] Li Y., Rapkin B.D. (2009). Classification and regression tree analysis to identify complex cognitive paths underlying quality of life response shifts: A study of individuals living with HIV/AIDS. J. Clin. Epidemiol..

[8] Rapkin B.D., Schwartz C.E. (2015). Distilling the essence of appraisal: A mixed methods study of people with multiple sclerosis. Qual. Life Res..

[9] Rapkin B., Weiss E., Chhabra R., Ryniker L., Patel S., Carness J., Adsuar R., Kahalas W., DeLaMarter C., Feldman I. (2008). Beyond satisfaction: Using the Dynamics of Care assessment to better understand patients’ experiences in care. Health Qual. Life Outcomes.

[10] Schwartz C.E., Michael W., Rapkin B.D. (2017). Resilience to health challenges is related to different ways of thinking: Mediators of physical and emotional quality of life in a heterogeneous rare-disease cohort. Qual. Life Res..

[11] Schwartz C.E., Powell V.E., Rapkin B.D. (2016). When global rating of change contradicts observed change: Examining appraisal processes underlying paradoxical responses over time. Qual. Life Res..

[12] Schwartz C.E., Zhang J., Rapkin B.D., Finkelstein J.A. (2018). Reconsidering the minimally important difference: Evidence of instability over time and across groups. Spine J..

[13] Finkelstein J.A. (2019). Measurement of appraisal is a valuable adjunct to the current spine outcome tools: A clinician’s perspective on the Rapkin and Schwartz commentary. Qual. Life Res..

[14] Turk D.C. (2004). Understanding pain sufferers: The role of cognitive processes. Spine J..

[15] Finkelstein J.A., Stark R.B., Lee J., Schwartz C.E. (2021). Patient factors that matter in predicting spine surgery outcomes: A machine learning approach. J. Neurosurgery Spine.

[16] Sniderman J., Stark R.B., Schwartz C.E., Imam H., Finkelstein J.A., Nousiainen M.T. (2021). Patient Factors That Matter in Predicting Hip Arthroplasty Outcomes: A Machine-Learning Approach. J. Arthroplast..

[17] Rapkin B.D., Schwartz C.E. (2019). Advancing quality-of-life research by deepening our understanding of response shift: A unifying theory of appraisal. Qual. Life Res..

[18] Schwartz C.E., Quaranto B.R., Rapkin B.D., Healy B.C., Vollmer T., Sprangers M.A.G. (2013). Fluctuations in appraisal over time in the context of stable versus non-stable health. Qual. Life Res..

[19] Rapkin B.D., Garcia I., Michael W., Zhang J., Schwartz C.E. (2017). Distinguishing appraisal and personality influences on quality of life in chronic illness: Introducing the quality-of-life Appraisal Profile version 2. Qual. Life Res..

[20] Rapkin B.D., Schwartz C.E. (2004). Toward a theoretical model of quality-of-life appraisal: Implications of findings from studies of response shift. Health Qual. Life Outcomes.

[21] Schwartz C.E., Stark R.B., Rapkin B.D. (2020). Capturing patient experience: Does quality-of-life appraisal entail a new class of measurement?. J. Patient-Rep. Outcomes.

[22] Schwartz C.E., Finkelstein J.A., Rapkin B.D. (2016). Appraisal assessment in patient-reported outcome research: Methods for uncovering the personal context and meaning of quality of life. Qual. Life Res..

[23] Ghogawala Z., Dziura J., Butler W.E., Dai F., Terrin N., Magge S.N., Coumans J.-V.C., Harrington J.F., Amin-Hanjani S., Schwartz J.S. (2016). Laminectomy plus Fusion versus Laminectomy Alone for Lumbar Spondylolisthesis. N. Engl. J. Med..

[24] Försth P., Ólafsson G., Carlsson T., Frost A., Borgström F., Fritzell P., Öhagen P., Michaëlsson K., Sandén B. (2016). A randomized, controlled trial of fusion surgery for lumbar spinal stenosis. NEJM.

[25] (2019). Alchemer. com.

[26] Fairbank J.C., Pynsent P.B. (2000). The Oswestry Disability Index. Spine.

[27] Ware J.E., Sherbourne C.D. (1992). The MOS 36-item short-form health survey (SF-36). I. Conceptual framework and item selection. Med. Care.

[28] Hays R.D., Sherbourne C.D., Mazel R.M. (1993). The rand 36-item health survey 1.0. Health Econ..

[29] Hays R.D., Morales L.S. (2001). The RAND-36 measure of health-related quality of life. Ann. Med..

[30] Ware J.E.J., Kosinski M., Dewey J.E. (2001). How to Score Version 2 of the SF-36 Health Survey (Standard & Acute Forms).

[31] Hays R.D., Prince-Embury S., Chen H. (1998). RAND-36 Health Status Inventory.

[32] Schwartz C.E., Stark R.B., Rapkin B.D. (2021). Creating idiometric short-form measures of cognitive appraisal: Balancing theory and pragmatics. J. Patient-Rep. Outcomes.

[33] Sangha O., Stucki G., Liang M.H., Fossel A.H., Katz J.N. (2003). The self-administered comorbidity questionnaire: A new method to assess comorbidity for clinical and health services research. Arthritis Care Res..

[34] Cohen J. (1992). A power primer. Psych. Bull..

[35] IBM (2019). IBM SPSS Statistics for Windows.

[36] Nilsdotter A.K., Lohmander L.S., Klässbo M., Roos E.M. (2003). Hip disability and osteoarthritis outcome score (HOOS)–validity and responsiveness in total hip replacement. BMC Musculoskelet. Disord..

[37] Schwartz C.E., Rapkin B.D., Sniderman J., Finkelstein J.A. (2022). Appraisal and patient-reported outcomes following total hip arthroplasty: A longitudinal cohort study. J. Patient-Rep. Outcomes.

[38] Burgess L.C., Arundel J., Wainwright T.W. (2019). The Effect of Preoperative Education on Psychological, Clinical and Economic Outcomes in Elective Spinal Surgery: A Systematic Review. Healthcare.

[39] Ljungqvist O., Scott M., Fearon K.C. (2017). Enhanced recovery after surgery: A review. JAMA Surg..

[40] Elsarrag M., Soldozy S., Patel P., Norat P., Sokolowski J.D., Park M.S., Tvrdik P., Kalani M.Y.S. (2019). Enhanced recovery after spine surgery: A systematic review. Neurosurg. Focus.

[41] Woby S.R., Roach N.K., Urmston M., Watson P.J. (2008). Outcome following a physiotherapist-led intervention for chronic low back pain: The important role of cognitive processes. Physiotherapy.

[42] Wynter-Blyth V., Moorthy K. (2017). Prehabilitation: Preparing patients for surgery. BMJ.

[43] Ditmyer M.M., Topp R., Pifer M. (2002). Prehabilitation in Preparation for Orthopaedic Surgery. Orthop. Nurs..

[44] Scheede-Bergdahl C., Minnella E., Carli F. (2019). Multi-modal prehabilitation: Addressing the why, when, what, how, who and where next?. Anaesthesia.

[45] Finkelstein J.A., Borowiec K., Schwartz C.E. (2022). Reconsidering Follow-Up in Spine Surgery Outcomes Research: An Analysis of Attrition and Necessary Length of Follow-Up.

[46] Edwards N.M., Varnum C., Overgaard S., Pedersen A.B. (2021). Impact of socioeconomic status on the 90-and 365-day rate of revision and mortality after primary total hip arthroplasty: A cohort study based on 103,901 patients with osteoarthritis from national databases in Denmark. Acta Orthop..

